# The Bristol Medico-Historical Society

**Published:** 1991-12

**Authors:** 


### NUMBER DEDICATED TO THE HISTORY OF MEDICINE

December 1991 Volume 106 (iv)

### The Bristol Medico-Historical Society

A notice appeared in the Bristol Medico-Chirurgical Journal of February 1986 proposing "the formation of a new society with an interest in the History of Medicine". The proposers "wished to see a small group, all of whom would be willing to give a paper from time to time", so that members would be stimulated to research their special interests; and instead of relying on invited speakers with expert knowledge on special subjects the society would be composed entirely of such people. And so it has turned out. With two papers at three meetings a year the members, initially limited to twenty four, have each given a paper. A four year cycle has been established. Pressure to enlarge the membership and a high attendance rate has encouraged us to have four meetings a year and admit eight new members. A selection of papers given to the society forms the basis of this number dedicated to the "History of Medicine".

